# Detection of Severe Acute Respiratory Syndrome Coronavirus 2 (SARS-CoV-2) including Variant Analysis by Mass Spectrometry in Placental Tissue

**DOI:** 10.3390/v14030604

**Published:** 2022-03-14

**Authors:** Marina Wierz, Beate Sauerbrei, Petra Wandernoth, Mark Kriegsmann, Rita Casadonte, Katharina Kriegsmann, Jörg Kriegsmann

**Affiliations:** 1 MVZ für Histologie, Zytologie und Molekulare Diagnostik Trier, 54296 Trier, Germany; m.wierz@patho-trier.de (M.W.); b.sauerbrei@patho-trier.de (B.S.); p.wandernoth@molekularpatho-trier.de (P.W.); 2 Institute of Pathology, University Hospital Heidelberg, 69120 Heidelberg, Germany; mark.kriegsmann@med.uni-heidelberg.de; 3 Translational Lung Research Centre Heidelberg, Member of the German Centre for Lung Research (DZL), 69120 Heidelberg, Germany; 4 Proteopath GmbH, 54926 Trier, Germany; r.casadonte@molekularpatho-trier.de; 5 Department of Hematology, Oncology and Rheumatology, University Hospital Heidelberg, 69120 Heidelberg, Germany; katharina.kriegsmann@med.uni-heidelberg.de; 6 Faculty of Medicine/Dentistry, Danube Private University, 3500 Krems-Stein, Austria

**Keywords:** SARS-CoV-2, FFPE, mass spectrometry, COVID-19, placenta

## Abstract

Among neonates, tested positive for SARS-CoV-2, the majority of infections occur through postpartum transmission. Only few reports describe intrauterine or intrapartum SARS-CoV-2 infections in newborns. To understand the route of transmission, detection of the virus or virus nucleic acid in the placenta and amniotic tissue are of special interest. Current methods to detect SARS-CoV-2 in placental tissue are immunohistochemistry, electron microscopy, in-situ hybridization, polymerase chain reaction (PCR) and next-generation sequencing. Recently, we described an alternative method for the detection of viral ribonucleic acid (RNA), by combination of reverse transcriptase-PCR and mass spectrometry (MS) in oropharyngeal and oral swabs. In this report, we could detect SARS-CoV-2 in formal-fixed and paraffin-embedded (FFPE) placental and amniotic tissue by multiplex RT-PCR MS. Additionally, we could identify the British variant (B.1.1.7) of the virus in this tissue by the same methodology. Combination of RT-PCR with MS is a fast and easy method to detect SARS-CoV-2 viral RNA, including specific variants in FFPE tissue.

## 1. Introduction

Severe acute respiratory syndrome coronavirus 2 (SARS-CoV-2), an enveloped positive-sense single-stranded RNA virus [1], has caused the current pandemic of severe respiratory infections, namely coronavirus disease 2019 (COVID-19) [2]. COVID-19 poses a challenge to global human health and public safety, as it leads to high morbidity and mortality [3], especially in patients with predisposing factors, such as high age, obesity, diabetes and hypertension [4].

Pregnancy is associated with an increased susceptibility for women to viral respiratory infections, but the impact of SARS-CoV-2 infection on pregnant patients, fetuses and neonates is poorly understood. Complications reported during pregnancy include preterm birth [5,6], spontaneous abortion [7], as well as fetal and neonatal death [8]. Moreover, SARS-CoV-2 infections and COVID-19 have been registered in newborns [9], also in cases where maternal infection could not be identified [10]. However, the main route of transmission has not been established.

Transmission of viral infections to neonates can occur through environmental sources (postpartum) or through vertical passage, from mother to fetus. In the case of vertical transmission, viruses are transmitted either in utero (intrauterine) through the placenta (antepartum), or in the birth canal during delivery (intrapartum). While most cases describe a postpartum transmission route of SARS-CoV-2 [11], vertical transmission seems to be a rare event [12,13,14].

Vertical transmission via the placenta is commonly observed in many DNA viruses, such as Cytomegalovirus and Herpes simplex virus, but also, RNA viruses can pass trans-placentally to the fetus. SARS-CoV-2 can infect placental tissue by targeting ACE2 and TMPRSS2, which are expressed by cells comprising the outermost layer of the placenta, the so-called villous syncytiotrophoblasts (ST) [15]. However, only few reports demonstrate a direct SARS-CoV-2 infection of the placenta and, thus, a possible vertical trans-placental transmission [16,17,18,19,20].

To further elucidate how SARS-CoV-2 is propagated from mother to fetus, detection and typing of the virus or viral nucleic acids in placental tissue is required. Current methods to detect the virus in the placenta include molecular and immunohistochemical assays. Besides standard RT-PCR to detect viral RNA [16,19,20,21], in-situ hybridization (ISH) [17,22] or immunohistochemistry (IHC) [18,19,23,24] were used to investigate the viral spike protein mRNA or the nucleocapsid protein, respectively. Furthermore, few studies performed ultrastructural analysis of SARS-CoV-2-infected placentas by electron microscopy [16,21,25,26].

Here, we describe an alternative method for the detection and typing of SARS-CoV-2 RNA in Formalin-fixed paraffin-embedded (FFPE) tissue. We report not only the positivity of SARS-CoV-2, but also the presence of the British (B.1.1.7) variant in placental and amniotic tissue, from a pregnant woman with COVID-19, thus, showing evidence for a possible trans-placental transmission of the virus. Understanding the biological behavior and the transmission route of the virus during pregnancy is important for the obstetrical management of pregnant women with SARS-CoV-2 infection.

## 2. Materials and Methods

### 2.1. Sample Collection

Samples were collected at the Institute of Pathology (Harzklinikum Dorothea Christiane Erxleben) in Quedlinburg. Both placental and amniotic tissues were obtained from a 27-year-old woman with monochorionic pregnancy at gestational week 20 (second trimester), which has tested positive for the British (B.1.1.7) variant of SARS-CoV-2. Tissues were fixed in buffered formalin, paraffin embedded, and the FFPE tissue blocks were sent to the Molecular Pathology Lab in Trier. Serial sections of each FFPE tissue block were taken and divided for routine H&E staining and RNA isolation.

### 2.2. Ribonucleic Acid (RNA) Extraction from FFPE Tissue

Semi-automated purification of total RNA from FFPE tissue was performed using the Maxwell^®^ RSC RNA FFPE Kit (Promega, ref. AS1440) following the manufacturer’s instructions. In brief, FFPE tissue sections were deparaffinized with mineral oil (2 min, 80 °C) and then transferred to 250 µL lysis buffer, centrifuged for phase separation and incubated for 15 min at 56 °C, followed by 1 h at 80 °C. After DNase I treatment (15 min, RT), the aqueous phase was loaded to a Maxwell^®^ RSC Instrument and RNA was semiautomatically isolated by paramagnetic particles. Next, 10 µL MS2 phage control included in the MassARRAY^®^ SARS-CoV-2 Panel (Material and Methods Section 2.3) was added to the lysis buffer to monitor RNA extraction.

### 2.3. SARS-CoV-2 Qualitative Detection and Variant Analysis by Mass Spectrometry (MALDI-TOF MS)

**Figure 1:**
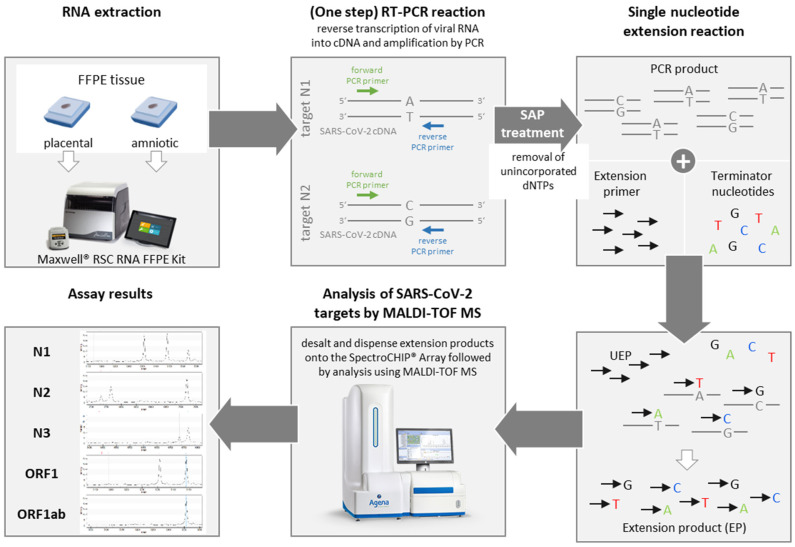
**Workflow and molecular principle of SARS-CoV-2 RNA detection in FFPE tissue by MALDI-TOF mass spectrometry.** RNA extraction from FFPE placental and amniotic tissue was performed using the Maxwell RSC RNA FFPE Kit (Promega) according to manufacturer’s instructions. Extracted viral RNA is then reverse transcribed into cDNA and amplified by a one-step RT-PCR reaction. The figure schematically depicts specific amplification of the SARS-CoV-2 targets N1 and N2. The forward primers are indicated in green, the reverse primers in blue. Thereafter, PCR products are treated with the shrimp alkaline phosphatase (SAP) enzyme to remove unincorporated nucleotides (dNTPs). Next, a single nucleotide extension reaction is performed, in which target-specific extension primers are elongated by a single mass-modified terminator nucleotide (A, T, C or G) complementary to the cDNA template sequence. The extension products (EP) are then analyzed by matrix-assisted laser desorption/ionization time-of-flight (MALDI-TOF) mass spectrometry (MS) using the MassARRAY system (Agena Bioscience) and mass spectra of the SARS-CoV-2-specific detected cDNA fragments are generated. In the absence of viral RNA, RT-PCR as well as single nucleotide extension reactions cannot be performed and consequently, no cDNA fragments are detected by MALDI-TOF analysis. EP: extension product; UEP: unextended extension primer.

Detection and variant analysis of SARS-CoV-2 RNA was performed by a multiplex (iPLEX^®^) assay procedure followed by matrix-assisted laser desorption/ionization time-of-flight (MALDI-TOF) mass spectrometry (MS) using the MassARRAY^®^ System (Agena Bioscience, Hamburg, Germany). The workflow and molecular principle are schematically depicted in Figure 1. Briefly, after reverse transcription (RT) of RNA into cDNA and amplification of SARS-CoV-2 target regions by PCR, unincorporated dNTPs are inactivated by shrimp alkaline phosphatase (SAP) followed by an extension PCR reaction, in which ‘extend’ primers specific for the amplified cDNA are elongated by a single mass-modified terminator nucleotide (single-base extension). The resultant extension products (analytes) are then desalted, transferred to a silicon chip with pre-spotted matrix material (SpectroCHIP^®^ Array), and loaded onto the MALDI-TOF mass spectrometer (MassARRAY Analyzer 4, Agena Bioscience). Following desorption and ionization of the analyte/matrix cocrystals using a UV-laser, the ionized DNA molecules/fragments are accelerated in an electrostatic field and separated by time-of-flight according to their molecular mass. Finally, data are processed and mass spectra displaying intensity and mass of the detected fragments are generated. Additionally, a final report is produced within the commercial software (MassARRAY Typer Analyzer Software v5.0.2, Agena Bioscience) including quality check and details on each analyzed SARS-CoV-2 target region.

**Figure 2:**
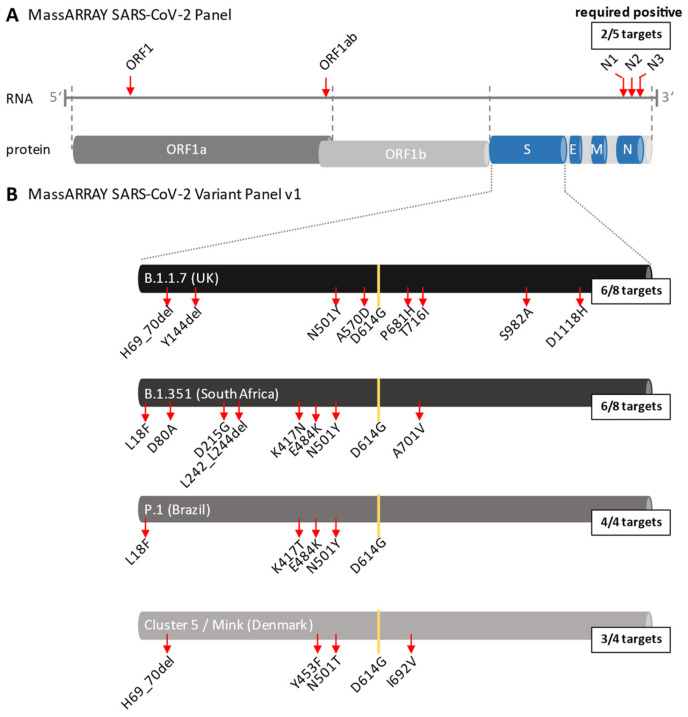
**Genomic targets of the MassARRAY SARS-CoV-2 Panels**. (**A**): The CE-IVD-certified MassARRAY SARS-CoV-2 Panel targets 5 genomic regions within the SARS-CoV-2 genome: ORF1, ORF1ab, N1, N2 and N3 (indicated by red arrows). The detection of at least 2 targets is required for the result “SARS-CoV-2 positive”. ORF, open reading frame; S, spike; E, envelope; M, membrane; N, nucleocapsid. (**B**): The MassARRAY SARS-CoV-2 Variant Panel v1 targets 20 specific mutations (indicated by red arrows) within the spike gene allowing the identification of 5 different SARS-CoV-2 variants. For detection of the British (B.1.1.7) variant at least 6 out of the 8 mutations H69_70 del, Y144del, N501Y, A570D, P681H, T716I, S982A, D1118H are required while for detection of the South African (B.1.351) variant at least 6 out of the 8 mutations L18F, D80A, D215G, L242_L244del, K417N, E484K, N501Y and A701V are required. The 4 mutations L18F, K417T, E484K and N501Y identify the Brazilian (P.1) variant and the 4 mutations H69_70del, Y453F, N501T and I692V allow the identification of the Danish (Mink) variant (3 out of 4 mutations required). The mutation D614G evolved very early during the pandemic spread in 2020 and is present in all variants.

The CE-IVD-certified MassARRAY^®^ SARS-CoV-2 Panel (Agena Bioscience, ref. 13279F), which includes 5 targets within the SARS-CoV-2 genome (N1, N2, N3, ORF1 and ORF1ab (Figure 2A), was used for the qualitative detection of SARS-CoV-2 RNA as described previously according to the manufacturer´s instructions [27].

For detection of the SARS-CoV-2 variants the Research Use Only (RUO) MassARRAY^®^ SARS-CoV-2 Variant Panel v1 (Agena Bioscience) was used. This panel includes 20 assays that are performed in two multiplexed reactions and that target specific mutations within the spike (S) gene of the SARS-CoV-2 genome for identification of the British (B.1.1.7), South African (B.1.351), Brazilian (P.1) and Danish (Cluster 5/Mink) variants (Figure 2B). The panel also contains a GAPDH assay that serves as an internal quality control (QC) for RNA extraction, RT-PCR, SAP and extension steps, as well as one assay probing the SARS-CoV-2 nucleocapsid (N) gene. The assay was performed according to the manufacturer’s protocol (Agena Bioscience, USG-CUS-151 R1.X1). Synthetic reference material with known mutations of the B.1.1.7 and B.1.351 variants was used as a positive control, whereas HPLC-grade water was used as negative control at each run. A final report (MassARRAY SARS-CoV-2 Variant Report-v1) listing QC status, calling of each individual target/mutation (detected or not detected) and the resulting/associated SARS-CoV-2 variant status was generated within the MassARRAY Typer Analyzer software. According to the reporting software, a positive result for the B.1.1.7 and B.1.351 variant is defined as 6 or more out of 8 positive mutation calls. Identification of the P.1 and Cluster 5/Mink variant requires 4 out of 4 and 3 out of 4 positive mutation calls, respectively (Figure 2B).

### 2.4. Reverse Transcription Real-Time- (RT-) Polymerase Chain Reaction (PCR)

Qualitative RT-PCR was performed using the EURORealTime SARS-CoV-2 Kit (EUROIMMUN, Ref. MP 2606-1000) on the LightCycler 480 II (Roche) system according to manufacturer’s instructions to confirm the result generated from the mass spectrometric analysis. The kit converts viral RNA into cDNA followed by amplification and fluorescence-based real-time detection of two defined regions within the ORF1ab- and N-genes of the SARS-CoV-2 genome. The kit also includes an internal RNA control (IC) as well as a positive control (PC) monitoring correct sample processing and amplification.

## 3. Results

### 3.1. Detection of SARS-CoV-2 RNA in Placental and Amniotic Tissue by MALDI-TOF

**Figure 3:**
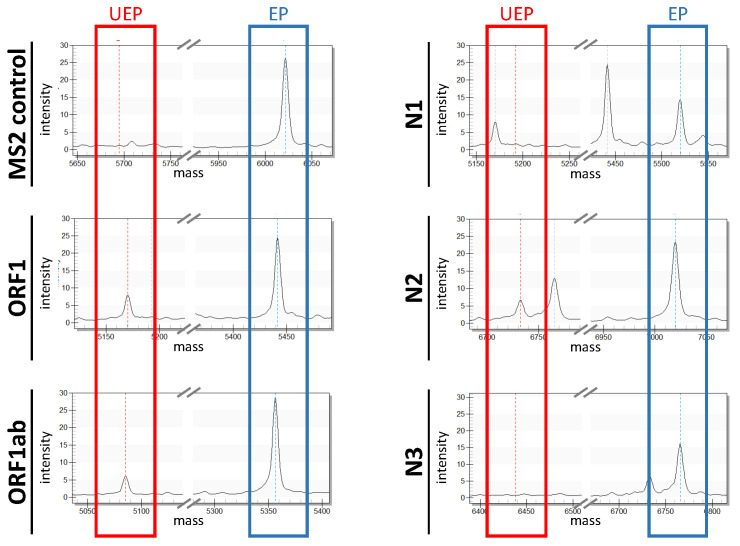
**Representative mass spectra of a positive sample analyzed by the CE-IVD-certified MassARRAY SARS-CoV-2 Panel.** The different mass spectra display intensity and mass (in daltons) of the SARS-CoV-2 -specific cDNA fragments ORF1, ORF1ab, N1, N2 and N3. Additionally, the mass spectrum of the MS2 phage control is depicted. The red box highlights the unextended extension primer peak, whereas the blue box highlights the peak corresponding to the extension product. UEP: unextended extension primer; EP: extension product.

**Figure 4:**
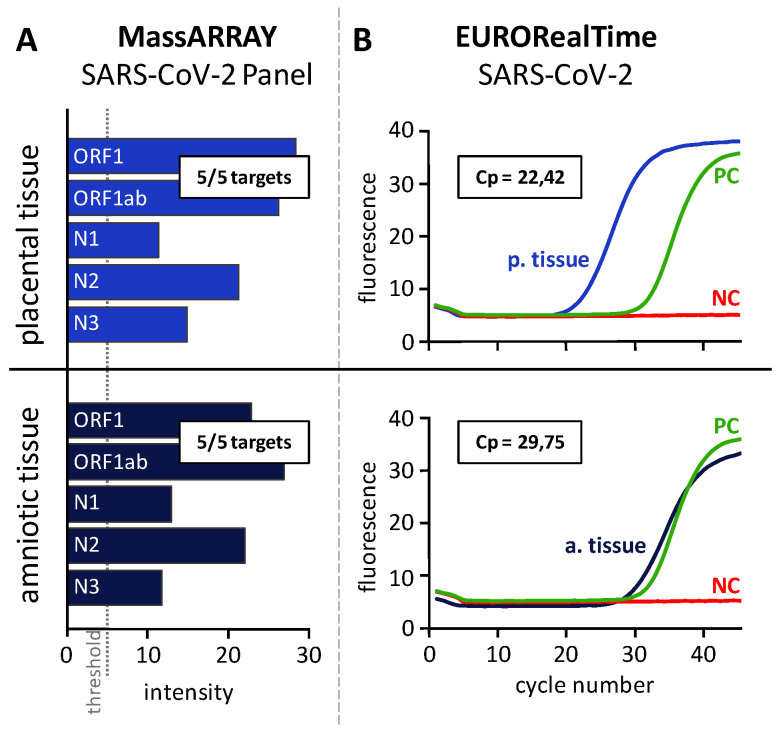
**Detection of SARS-CoV-2 RNA in placental and amniotic tissue.** Placental and amniotic tissue were analyzed by MALDI-TOF MS using the CE-IVD-certified MassARRAY SARS-CoV-2 Panel (**A**) and by RT-PCR using the EURORealTime SARS-CoV-2 Kit (**B**). (**A**): The plots display the intensity of the peaks corresponding to the 5 targets ORF1, ORF1ab, N1, N2 and N3 included in the CE-IVD-certified MassARRAY SARS-CoV-2 Panel. The intensity threshold considering a peak as detected is 5. All 5 SARS-CoV-2 targets were detected in both placental and amniotic tissue. (**B**). The graphs show the fluorescence intensity (*y*-axis) for each cycle (*x*-axis) of the real-time amplification of the SARS-CoV-2 -specific target regions (amplification curve). The amplification curve of the positive control (PC) is highlighted in green, the one of the negative control (NC) in red. The crossing point (Cp) value is indicated for both placental tissue (p.tissue) and amniotic tissue (a.tissue).

Both samples successfully passed the quality check, as demonstrated by detection of the MS2 phage control (Figure 3). As expected, in the positive control (PC), all five SARS-CoV-2 targets (N1, N2, N3, ORF1 and ORF1ab) could be identified, whereas the negative control (NC) did not show any viral cDNA fragments (data not shown). In both placental and amniotic tissues, all targets of the SARS-CoV-2 genome could be detected with a peak intensity higher than 5, demonstrating the presence of SARS-CoV2 RNA in these tissues. The positivity of the tissue specimens could be confirmed by RT-PCR, as they all showed amplification of the two genomic regions, ORF1ab and N, with lower Cp values when compared to the positive control (PC, mean Cp PC = 31,7, mean Cp placental tissue 1 = 22.5, mean Cp amniotic tissue = 29.8; Figure 4).

### 3.2. Identification of the British (B.1.1.7) Variant of SARS-CoV-2 in FFPE Tissue by MALDI-TOF

**Figure 5:**
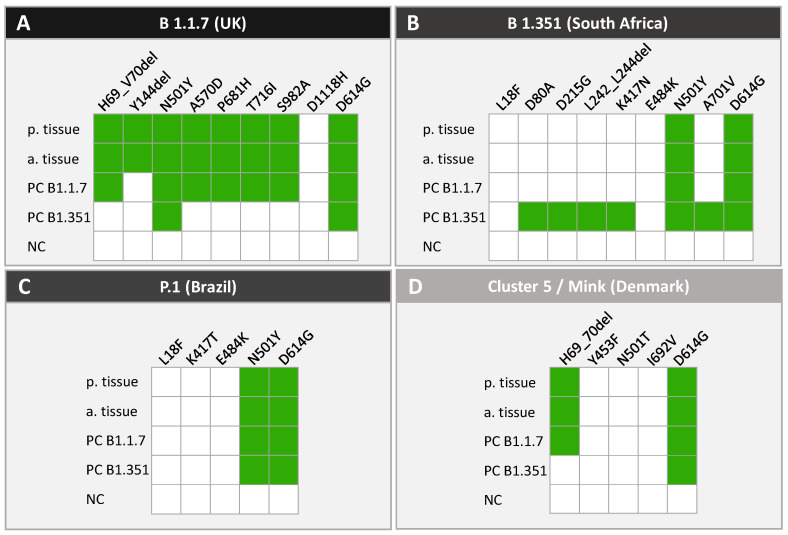
**SARS-CoV-2 variant analysis of amniotic and placental tissue by MALDI-TOF MS.** Placental and amniotic tissue were analyzed by MALDI-TOF MS using the MassARRAY SARS-CoV-2 Variant Panel v1. The figure shows the specific mutations within the SARS-CoV-2 spike gene that are assigned to the different variants B 1.1.7 (**A**), B 1.351 (**B**), P.1 (**C**) and Cluster 5/Mink (**D**). Detected mutations in placental tissue (p.tissue), amniotic tissue (a.tissue), positive controls (PC) B 1.1.7 and B 1.351 and negative control (NC) are illustrated by green-colored squares.

As we could detect a clear invasion of SARS-CoV-2 in the placental and amniotic tissue, we analyzed the tissues with the same methodology for SARS-CoV-2 variants, using the MassARRAY SARS-CoV-2 Variant Panel, which targets 20 specific mutations within the spike gene (Figure 2B). Both samples passed the endogenous quality control (GAPDH and N gene positive call, data not shown). We detected the mutation D614G, which has been described as the first spike mutation altering the fitness of SARS-CoV-2 [28], as well as seven additional mutations (A570D, H69_V70del, N501Y, P681H, S982A, T716I, Y144del), which are associated with the British (B.1.1.7) variant. The B.1.1.7-associated mutation D1118H was not detected, neither in placenta nor in amniotic tissue. Furthermore, mass spectrometry did not reveal any mutation specific for the South African (B.1.351), Brazilian (P.1) or Danish (Cluster 5/Mink) variants (Figure 5).

## 4. Discussion

Viral infections of the placenta include infection by Cytomegalovirus, Hepatitis Viruses, Varicella Zoster Virus, Parvovirus B19, Human Immunodeficiency Virus, Rubella Virus, and Herpes simplex Virus [29]. Today, viral infection of the placental tissue by SARS-CoV-2 is of special interest, since clinical information about the incidence of vertical transmission of SARS-CoV-2 is limited to date [30]. Recent reports from 262 deliveries showed evidence of SARS-CoV-2 infection of the children in 1–11% of cases [13,31,32].

We show clear invasion of the placenta with SARS-CoV-2, suggesting prepartum (transplacental) transmission. However, transmission routes from mother to infant remain poorly understood. Raschetti and colleagues performed a systematical meta-analysis on 74 publications, including a total of 176 neonates tested positive for SARS-CoV-2. The authors estimated that, among all published cases of neonates tested positive for SARS-CoV-2, approximately 70% acquired their infection through postpartum transmission (i.e., environmental exposure) and 30% likely became infected either through prepartum (i.e., in utero transplacental) or intrapartum (i.e., in the birth canal during delivery) mechanisms. Among these, 5.7% were stated to have confirmed prepartum infection [11]. Interestingly, SARS-CoV-2 may be detected in the placental tissue, but not in the fetus in some cases [16,20], and some authors reported that viral load was found higher in the placental tissue than in amnion fluid and maternal or neonatal blood [19]. This might be due to the high expression of SARS-CoV-2 receptors in the placental tissue [33]. The body of evidence on the clinical impact of SARS-CoV-2 in pregnancy becomes broader. Most reports do not report higher rates of preterm birth and intrauterine growth restriction in women with SARS-CoV-2 infection/COVID-19, but severe maternal comorbidity and perinatal death has rarely been reported [34,35]. Preventive measures for pregnant women include personal hygiene, proper nutrition and social distancing [35].

Histological findings of SARS-CoV-2 infection in the placenta were reported to be non-specific and include evidence of maternal–fetal vascular malperfusion, villous agglutination, subchorionic thrombi, marked increase in perivillous fibrin deposition and inflammatory changes, such as villitis, intervillositis and chorioamnionitis [33,36]. Thus, evidence of SARS-CoV-2 infection needs additional molecular methods, beyond pure histopathological evaluation, and diagnostic criteria were proposed to suggest intrauterine transmission. These criteria include demonstration of SARS-CoV-2 in the fetal-derived cells of the placenta (e.g., syncytiotrophoblasts, Hofbauer cells, villous stromal, and endothelial cells), using molecular pathology techniques, such as immunohistochemistry or RNA in-situ hybridization in mother–neonate dyads testing positive for SARS-CoV-2 [37]. But the range of techniques that have been used to detect SARS-CoV-2 in placental tissue is much broader and not only includes immunohistochemistry [16,19,21,33,37,38], or in-situ hybridization [17,33,37], but also RT-PCR [16,19,20,21], next-generation sequencing [16] and electron microscopy [16,20,25].

Previously, we have shown that genetic mutations in tumor tissue [39] and human papilloma virus [40] can be detected by mass spectrometry. Furthermore, we have shown that SARS-CoV-2 can be identified in oro- and nasopharyngeal swabs by mass spectrometry [27]. In the present report, we used mass spectrometry, for the first time, for identification of SARS-CoV-2 infection and for variant typing in placental tissue and confirmed the results by RT-PCR.

Both technologies, mass spectrometry and RT-PCR, have advantages and disadvantages. Time and costs are similar, with slightly longer hands-on-time (45 min vs. 15 min) and time-to-results (8 h vs. 5 h) with the MassARRAY system, as compared to RT-PCR. Costs for material are also comparable and in the range of EUR 10–15 for both assays. The MassARRAY can process up to 96 samples per batch and our RT-PCR assay can process up to 44 samples. The main advantage of the MassARRAY system, as compared to RT-PCR, is that samples do not need to run in duplicates using the MassARRAY, as multiple genomic regions are targeted simultaneously (five vs. two targets). Moreover, the mass spectrometric system performs not only detection, but also rapid and reliable typing of SARS-CoV-2. Hence, the mass spectrometric system is fast, reliable, cost-effective and provides important clinical and epidemiological information, which makes it a good alternative for high-throughput testing of samples collected from swabs and FFPE tissue.

## Data Availability

Not applicable.
